# Supplementary material for: Site-specific machine learning predictive fertilization models for potato crops in Eastern Canada
Source: PLoS One. 2020 Aug 7;15(8):e0230888. doi: 10.1371/journal.pone.0230888 (PMC7413527; doi:10.1371/journal.pone.0230888)
Supplement: S1 Table — (DOCX) [file pone.0230888.s001.docx]

**S1 Table: Description of the data set used for the marketable yield models**

| **Study year** | **Number of trials** | **Number of samples** | **Percentage (%)** | **Minimum number of blocks** | **Maximum number of blocks** | **Minimum number of treatments** | **Maximum number of treatments** |
| --- | --- | --- | --- | --- | --- | --- | --- |
| 1979 | 1 | 10 | 0.2 | 3 | 3 | 4 | 4 |
| 1980 | 1 | 10 | 0.2 | 3 | 3 | 4 | 4 |
| 1981 | 3 | 30 | 0.5 | 6 | 6 | 4 | 4 |
| 1987 | 1 | 8 | 0.1 | 2 | 2 | 4 | 4 |
| 1993 | 6 | 144 | 2.4 | 3 | 3 | 27 | 27 |
| 1994 | 3 | 84 | 1.4 | 3 | 3 | 10 | 10 |
| 1995 | 3 | 81 | 1.4 | 3 | 3 | 9 | 9 |
| 1996 | 8 | 258 | 4.4 | 6 | 6 | 11 | 11 |
| 1997 | 8 | 306 | 5.2 | 6 | 6 | 22 | 22 |
| 1998 | 14 | 280 | 4.7 | 6 | 6 | 5 | 5 |
| 1999 | 18 | 431 | 7.3 | 6 | 6 | 14 | 14 |
| 2000 | 8 | 184 | 3.1 | 6 | 6 | 5 | 5 |
| 2001 | 8 | 152 | 2.6 | 4 | 4 | 5 | 5 |
| 2002 | 8 | 183 | 3.1 | 4 | 4 | 11 | 11 |
| 2003 | 21 | 348 | 5.9 | 4 | 4 | 10 | 10 |
| 2004 | 11 | 267 | 4.5 | 3 | 3 | 11 | 11 |
| 2005 | 20 | 574 | 9.7 | 4 | 4 | 11 | 11 |
| 2006 | 10 | 186 | 3.1 | 3 | 3 | 10 | 10 |
| 2007 | 15 | 297 | 5.0 | 3 | 3 | 12 | 12 |
| 2008 | 6 | 107 | 1.8 | 3 | 3 | 7 | 7 |
| 2009 | 6 | 118 | 2.0 | 5 | 5 | 8 | 8 |
| 2010 | 8 | 154 | 2.6 | 4 | 4 | 8 | 8 |
| 2011 | 13 | 261 | 4.4 | 4 | 4 | 8 | 8 |
| 2012 | 13 | 268 | 4.5 | 4 | 4 | 8 | 8 |
| 2013 | 16 | 344 | 5.8 | 4 | 4 | 8 | 8 |
| 2014 | 25 | 535 | 9.0 | 4 | 4 | 10 | 10 |
| 2015 | 2 | 33 | 0.6 | 3 | 3 | 6 | 6 |
| 2016 | 5 | 48 | 0.8 | 3 | 3 | 6 | 6 |
| 2017 | 12 | 212 | 3.6 | 3 | 3 | 8 | 8 |
| Total | 273 | 5913 | 100.0 |  |  |  |  |

Similar tables are reproducible for the tuber-size balances and specific gravity models by using the codes (file 1.3) available at <https://git.io/JvYxd>.
