# Supplementary material for: Site-specific machine learning predictive fertilization models for potato crops in Eastern Canada
Source: PLoS One. 2020 Aug 7;15(8):e0230888. doi: 10.1371/journal.pone.0230888 (PMC7413527; doi:10.1371/journal.pone.0230888)
Supplement: S2 Table — (DOCX) [file pone.0230888.s002.docx]

**S2 Table: Description of the modeling data sets per trial type**

**A. Marketable yield**

| **Trial type** | **Number of samples** | **Number of trials** | **Percentage** | **Minimum N dosage** | **Maximum N dosage** | **Minimum P_2_O_5_ dosage** | **Maximum P_2_O_5_ dosage** | **Minimum K_2_O dosage** | **Maximum K_2_O dosage** |
| --- | --- | --- | --- | --- | --- | --- | --- | --- | --- |
|  |  |  | **(%)** | **(kg ha^-1^)** | | | | | |
| **K** | 936 | 45 | 15.8 | 80 | 260 | 75 | 240 | 0 | 420 |
| **N** | 3,068 | 151 | 51.9 | 0 | 250 | 88 | 250 | 57 | 270 |
| **NPK** | 591 | 16 | 10.0 | 0 | 225 | 0 | 300 | 0 | 300 |
| **P** | 1,300 | 60 | 22.0 | 0 | 260 | 0 | 300 | 0 | 270 |
| **NA** | 18 | 1 | 0.3 | 218 | 218 | 110 | 110 | 55 | 55 |
| **Total** | **5,913** | **273** | **100.0** |  |  |  |  |  |  |

**B. Tuber size balances**

| **Trial type** | **Number of samples** | **Number of trials** | **Percentage** | **Minimum N dosage** | **Maximum N dosage** | **Minimum P_2_O_5_ dosage** | **Maximum P_2_O_5_ dosage** | **Minimum K_2_O dosage** | **Maximum K_2_O dosage** |
| --- | --- | --- | --- | --- | --- | --- | --- | --- | --- |
|  |  |  | **(%)** | **(kg ha^-1^)** | | | | | |
| **K** | 901 | 43 | 19.8 | 80 | 220 | 75 | 200 | 0 | 300 |
| **N** | 2,378 | 122 | 52.2 | 0 | 250 | 100 | 216 | 57 | 270 |
| **NPK** | 363 | 9 | 8.0 | 0 | 225 | 0 | 300 | 0 | 300 |
| **P** | 897 | 33 | 19.7 | 110 | 210 | 0 | 300 | 0 | 270 |
| **NA** | 18 | 1 | 0.4 | 218 | 218 | 110 | 110 | 55 | 55 |
| **Total** | **4,557** | **208** | **100.0** |  |  |  |  |  |  |

**C. Tuber specific gravity**

| **Trial type** | **Number of samples** | **Number of trials** | **Percentage** | **Minimum N dosage** | **Maximum N dosage** | **Minimum P_2_O_5_ dosage** | **Maximum P_2_O_5_ dosage** | **Minimum K_2_O dosage** | **Maximum K_2_O dosage** |
| --- | --- | --- | --- | --- | --- | --- | --- | --- | --- |
|  |  |  | **(%)** | **(kg ha^-1^)** | | | | | |
| **K** | 880 | 42 | 20.7 | 80 | 260 | 75 | 240 | 0 | 420 |
| **N** | 1,956 | 117 | 46.0 | 0 | 250 | 88 | 215 | 57 | 270 |
| **NPK** | 410 | 16 | 9.6 | 0 | 225 | 0 | 300 | 0 | 300 |
| **P** | 990 | 38 | 23.3 | 110 | 260 | 0 | 300 | 0 | 270 |
| **NA** | 18 | 1 | 0.4 | 218 | 218 | 110 | 110 | 55 | 55 |
| **Total** | **4,254** | **214** | **100.0** |  |  |  |  |  |  |
