# Supplementary material for: Site-specific machine learning predictive fertilization models for potato crops in Eastern Canada
Source: PLoS One. 2020 Aug 7;15(8):e0230888. doi: 10.1371/journal.pone.0230888 (PMC7413527; doi:10.1371/journal.pone.0230888)
Supplement: S3 Table — (DOCX) [file pone.0230888.s003.docx]

**S3 Table.** **Classification of preceding crops [7]**

| **Metal-level** | **Preceding crop** |
| --- | --- |
| Small grain | Oat, oat and mustard, wheat, canola, cereal, mustard, white mustard, barley, barley and mustard, sunola, and cereal |
| High-residue crops | Grain corn, pearl millet, rye |
| Legume | Perennial legumes: birdsfoot trefoil, alfalfa, legume mix, clover, ryegrass, and clover |
| Grassland | Orchard grass, ryegrass, and cereal |
| Low-residue crops | Annual low-residue crops: broccoli, cabbage, Chinese cabbage, beans, strawberry, silage corn, onion, pea, potato, potato followed by green manure, buckwheat, soybean |
