# Supplementary material for: Site-specific machine learning predictive fertilization models for potato crops in Eastern Canada
Source: PLoS One. 2020 Aug 7;15(8):e0230888. doi: 10.1371/journal.pone.0230888 (PMC7413527; doi:10.1371/journal.pone.0230888)
Supplement: S4 Table — (DOCX) [file pone.0230888.s004.docx]

**S4 Table.** **Centroids of soil textural classes derived from the Quebec soils data set [57]**

| **Textural class** | **Sand (%)** | **Silt (%)** | **Clay (%)** |
| --- | --- | --- | --- |
| **Coarse sand** | 93.8 | 3.3 | 2.9 |
| **Sand** | 92.7 | 4.4 | 2.9 |
| **Fine sand** | 92.3 | 4.6 | 3.1 |
| **Very fine sand** | 92.9 | 4.4 | 2.7 |
| **Coarse loamy sand** | 82.5 | 11.3 | 6.2 |
| **Loamy sand** | 82.5 | 12.1 | 5.4 |
| **Fine loamy sand** | 81.4 | 14.4 | 4.2 |
| **Very fine loamy sand** | 80.7 | 11.2 | 8.1 |
| **Coarse sandy loam** | 66.2 | 20.4 | 13.4 |
| **Sandy loam** | 64.5 | 25.0 | 10.5 |
| **Fine sandy loam** | 60.4 | 31.6 | 8.0 |
| **Very fine sandy loam** | 60.2 | 32.8 | 7.0 |
| **Loam** | 42.4 | 41.1 | 16.5 |
| **Silty loam** | 18.2 | 67.0 | 14.8 |
| **Silt** | 6.0 | 88.1 | 5.9 |
| **Sandy clay loam** | 55.7 | 19.9 | 24.4 |
| **Clay loam** | 29.5 | 38.4 | 32.1 |
| **Silty clay loam** | 7.8 | 58.1 | 34.1 |
| **Sandy clay** | 50.2 | 11.2 | 38.6 |
| **Silty clay** | 2.7 | 48.1 | 49.2 |
| **Clay** | 11.9 | 34.9 | 53.2 |
| **Heavy clay** | 1.3 | 23.9 | 74.8 |
