# Supplementary material for: Site-specific machine learning predictive fertilization models for potato crops in Eastern Canada
Source: PLoS One. 2020 Aug 7;15(8):e0230888. doi: 10.1371/journal.pone.0230888 (PMC7413527; doi:10.1371/journal.pone.0230888)
Supplement: Attachment — Submitted filename: Response to Reviewers.docx [file pone.0230888.s006.docx]

Response to reviewers

**Dear editor and reviewer(s),**

We revised the manuscript (PONE-D-20-06893) entitled “Site-specific machine learning predictive fertilization models for potato crops in Eastern Canada” according to the reviewers’ comments. We thank the referees for their careful and constructive reviews.

The responses are detailed according to the progression of the reviewer’s notes.

Thank you again for your consideration of our revised manuscript.

Best regards,

Serge-Étienne Parent, Eng, PhD

Assistant Professor, Laval University

Faculty of Agriculture and Food Sciences

Department of Soils and Food Engineering

2425, Rue de l’agriculture

Québec, Quebec, G1V 0A6, Canada

Review Comments to the Author

Reviewer #1

“The authors made an interesting work in assessing site-specific learning predictive fertilization models for potato crops in Eastern Canada. They used multiple data and variables trying to assess different prediction methods regarding tuber yield and potato quality (also N, P, K requirements for potato yield and quality). However, the data are not presented clearly and in detail (no descriptive statistics) so we don't know if they are the appropriate for the current models. The authors tried to combine data from different time periods (from the last 40 years) something that can also be problematic and needs more clarification. Finally, they tried to assess multiple things at the same time. This confuses the reader and perplexes the understanding of the goals and the scope of the paper. Therefore, we believe that the manuscript needs major revisions before it could be accepted for publication”. The authors should consider the following:

1. Please provide detailed descriptive statistics for all the data that you used. Also provide measure of units, small description, methods of analysis and time (date) of measurements for your data. The current tables in the manuscript are more confusing than helpful.

**Answer**. We have provided several statistics for the data set by adding new tables (Table 1 and S1–S2 Tables). The repartitions of our samples are detailed in relation to target variables, the study year and the fertilizer tested (N, P, K or NA). The minimum and maximum number of blocks and treatments as well as fertilizer limits are presented. Data of the four trials used for model interpretation are gathered in a single table (Table 4). The table presenting the centroids of soil texture classes has been moved to supplementary information (S4 Table). We have provided measure of units where missing. It is also clearly specified that all the measurements are referring to the study year except for the weather indices which used data through the five seasons preceding the season of the study. This should improve the clarity of the manuscript.

2. The 273 field experiments that you use extend in a very long time period (from 1979 to 2017). Are you sure that the data from these experiments are consistent (e.g. same methods of collecting and measuring the parameters) so to be used in data analysis?

**Answer**. Fertilization trials were indeed conducted over a time span of four decades (1979–2017). We have described experimental procedure for all the trials of the data set. Although agricultural practices, soil conditions and analytical techniques have undergone substantial changes over time, Valkama et al. [101] has shown that the differences between old and recent experiments in yield responses are not statistically important. Moreover, where the analytical techniques for the same element differed, correlation equations were available to converting to one technique before data analysis. It is the case for soil carbon converted from Walkley-Black to Leco CNS (equation 1) in Basic soil composition section, soil pH processed with CaCl_2_ converted to pH water (equation 3) in Soil pH section, P-Bray-2 converted to P-Mehlich-3 (equation 4) in Soil Mehlich-3 extractable elements section of the Methodology. Since there were similarities in experimental procedures and ability to uniformly convert measurement methods, we found that the data set could be used for machine learning. We added this comment to the discussion.

3. How are the field experiments spread throughout the years? Provide at least some kind of classification with percentages (1979–1989 20%, 1990–1999 30% etc.).

**Answer**. We added new tables which clearly summarize the information (Table 1 and tables in S1–S2 Tables).

4. It is unclear how you combined the data from tables 1, 2, 3, 4, 5 and 6. For example, you may have data from a field experiment that happened 40 years ago and weather historical data from 5 years ago (line 222). Can you use them together? Also, please provide the time (date) of the collection and analysis of the soil profiles (and soil samples)? Are they consistent with the timeframe of the field experiments and weather data?

**Answer**. The new structure of the tables is clearer. The previous (the old) Table 1 was intended to summarize the data set but indeed did not do so sufficiently. The previous Tables 2 and 4 together gathered global information of trials used for model interpretation. The previous Table 3 (now moved in Supplementary Information) assigned texture centroids values to missing observations. The previous Tables 5 and 6 presented different formula to compute values of some predictive variables.

All the tables have been restructured:

The old Table 1 has been amended with new statistics provided,

the old Table 5 has been deleted since the equation 2 presents general formula to compute isometric log-ratios,

the old Table 6 is maintained but now as Tables 2,

the old Table 3 has been moved to supplementary information (S4 Table),

the old Tables 2 and 4 have been merged in the new Table 4 presenting model interpretation trials.

Data are used together only for modeling (training and testing the models). For interpretation, trials are used separately, one for estimating optimal doses and the others (1 N trial, 1 P trial and 1 K trial) to assess the behaviour of each model when considering the tested element. So, data corresponding to these trials are not merged to be used together.

For the question about time, the analyzes and measurements of each trial data were carried out during the study year. Weather data are averaged through five-years preceding the study year. We have specified these important details in the methodology.

5. You have the geographical coordinates from each experiment. It would be helpful if you provide a map with the locations of the experiments.

**Answer**. Such a map has been included.

6. You say that you added data from another 17 trials (line 124), in the 273 field experiments. How many are the field experiments overall and exactly how many are the samples for each parameter used in the models?

**Answer**. The new structure of the Methodology also removes this ambiguity. The 17 trials are among the 273. The table showing data per year of experiments (S1 Table) presents the 17 trials (5 in 2016 and 12 in 2017). The Table 1 and tables in S1–S2 Tables show the number of field experiments overall, and more specific statistics. The new Table 5 which summarizes the parameters (Results section) gives the number of samples for each model.

7. There seem to be a conflict between the objectives of the study. In lines 21-24 you say that you want to predict tuber yield and quality, whereas in lines 95-97 you state that you want to compare the performance of ML in predicting N,P,K requirements for potato yield and quality. Please make totally clear what you try to achieve. Also, try to keep it simple and to the point. Too much goals, confuse and distract.

**Answer**. The objective of the project has been amended in the Abstract to consider what we did specifically during the analysis (stated in the Introduction): The objective of this study was to determine an optimal model predicting nitrogen, phosphorus and potassium requirements for high tuber yield and quality as impacted by weather, soils and land management variables.

8. Please change "neuronal networks" to "neural networks" (e.g. In line 27 and line 284)

**Answer**. Changed.

9. What type of random cross validation did you use? (e.g. leave one out, k-fold, … how many folds?)

**Answer**. We have used a k-fold cross-validation specifically 5-fold cross-validation for all the models. This detail is now provided.

10. Check the "Error! Reference source not found" error

**Answer.** Corrected.

11. The tuning parameters (lines 361–374) are in the wrong place (4.1 Feature importance). Please place them in a separate section.

**Answer**. The tuning parameters are now presented in a separate section (Model tuning parameters).

12. Images in manuscript are not clear. They have a very low quality.

**Answer**. We have edited all the figures and ensured that they meet PLOS requirements using the PACE digital diagnostic tool (<https://pacev2.apexcovantage.com/>).

13. Too long image captions. *e.g* in lines 399–401 there is no need to explain what MAE, RMSE etc. are.

**Answer**. The images captions have been amended. We hope that the readers can refer to the definitions of the abbreviations given earlier through the manuscript.

14. In line 434 too many "and". Please rephrase.

**Answer**. The sentence has been deleted.

15. Please mention the prediction method/s that the feature importance (line 349) belongs.

**Answer**. The prediction method is now mentioned in the first sentence of the section:

*The feature importance, computed using the ExtratreesRegressor function, revealed that the N fertilizer dose was by far the most informative feature in the marketable yield prediction models, followed by soil type, air temperature, length of growing season and soil texture.*

We have also specified the method in the Methodology (Selection of features section).

16. In line 560, the discrepancies in scores between testing and training data shows problems of overfitting or underfitting?

**Answer**. When a model performs highly on the training set but poorly on the test set, this is known as overfitting. It is essentially creating a model that knows the training set very well but cannot be applied to new problems. Since the models performed well with the training data but weakly at testing, it is an overfitting. We have deleted the expression ‘*or underfitting*’ to remove the ambiguity.

17. The text that you use for the hyperparameters/parameters is confusing (lines 361–374). Please also provide a table/s with the hyperparameters/parameters of the models, a small description, and the values that you used.

**Answer**. The new section added (Model tuning parameters) provides more information.

Finally, after proofreading, your questions and comments helped us significantly improve the manuscript.
