# Supplementary material for: Site-specific machine learning predictive fertilization models for potato crops in Eastern Canada
Source: PLoS One. 2020 Aug 7;15(8):e0230888. doi: 10.1371/journal.pone.0230888 (PMC7413527; doi:10.1371/journal.pone.0230888)
Supplement: Attachment — Submitted filename: Response to Reviewers.docx [file pone.0230888.s007.docx]

Response to reviewers

**Dear editor and reviewer(s),**

We revised the manuscript (PONE-D-20-06893) entitled “Site-specific machine learning predictive fertilization models for potato crops in Eastern Canada” according to one reviewer comments. We thank the referees for their careful and constructive reviews.

Thank you again for your consideration of our revised manuscript.

Best regards,

Serge-Étienne Parent, Eng, PhD

Assistant Professor, Laval University

Faculty of Agriculture and Food Sciences

Department of Soils and Food Engineering

2425, Rue de l’agriculture

Québec, Quebec, G1V 0A6, Canada

Review Comments to the Author

Reviewer #2: The author has improved this work well under the comments of the first external review expert. The article is very informative and becomes more meaningful. The data is also supplemented. The article uses multiple statistical models and also adds a description. It was explained. However, we believe that there are several points in this article, which we hope can be improved:
Although the 40-year change mentions the improvement of the measurement method, etc., it is possible to discuss more about whether the variety has the property of preferring a certain fertilizer or the relationship between the varieties.

**Answer**. We have amended the discussion about the selection of predictive features in the last paragraph of the 5.1 section (Selection of features):

The relative importance of a variable in a model is related to its effect on the output through its gradient in the data set. Hence the predominance of N doses, and P and K doses to some extent, could have been caused by the origin of the data set, which is a collection of fertilizer trials where large gradients of doses are found by design. This study did not address fertilizer source and timing of application. While Marouani et al. [122] found equivalency of ammonium nitrate (33.5% N), urea (46% N), NP fertilizer (33% N – 14% P2O5) and NPK fertilizer (27%N – 5% P2O5 – 5% K2O), Petropoulos et al. [123] found that the form of the fertilizer (ammonium sulfate, ammonium sulfate + zeolite, manure, slow release N fertilizer with urease inhibitor) and the cultivar (Kennebec and Spunta) may affect yield and chemical composition of potato tubers, affecting the end use of the product. Flis [124] reported that the peculiarities of potato cultivar, plant root structure, and timing of nutrient uptake impact on the selection of a site-specific fertilization regime. Trehan et al. [125] showed that some cultivars exhibit strong symptoms of N, P and K deficiencies compared to others. Potato cultivars may sustain leaf development and nutrient uptake while maintaining maximum tuber growth rates to reach higher final tuber yields with contrasting nutrient requirements [126]. Differential effects of cultivar and fertilizer on tuber yield have also been reported by Daoui et al. [127]. In a previous study, Coulibali et al. [128] found that genetic traits were not compelling to set apart clusters of cultivar based on N, P, K, Mg and Ca compositions of diagnostic leaves. The cultivar effect was thus excluded from the present study to keep models parsimonious. In our analysis, we focused on the gradients of N, P and K doses while keeping the other site-specific factors constant. Nevertheless, predictive feature such as biotic factors (length of growing season, preceding crop and seeding density), could also be predicted and optimized by the models with respect to tuber yield and quality.

After proofreading, this suggestion helped us significantly improve the section and the manuscript.
